# Effect of a goal-directed sedation nursing intervention bundle on comfort and compliance in children undergoing bronchoalveolar lavage under topical anesthesia with procedural sedation: a randomized controlled trial

**DOI:** 10.3389/fmed.2026.1852309

**Published:** 2026-06-10

**Authors:** Xi Luo, Huafu Zhang

**Affiliations:** Nursing Department, Ma Chang Endoscopy Center, Children’s Hospital, Tianjin University/Tianjin Children’s Hospital, Tianjin, China

**Keywords:** bronchoalveolar lavage, children, comfort, compliance, goal-directed sedation, local anesthesia, nursing intervention

## Abstract

**Objective:**

To assess the impacts of goal-directed sedation nursing intervention on comfort and compliance in children undergoing bronchoalveolar lavage (BAL) under topical anesthesia with procedural sedation.

**Methods:**

A total of 168 children undergoing BAL under topical anesthesia with procedural sedation were randomly assigned to a study group and a control group. After post-randomization exclusions, the final analyzed sample comprised 82 and 81 participants, respectively. The intervention period was from November 2024 to October 2025. Seven outcome measures were assessed: (1) comfort level (primary outcome); (2) procedure compliance; (3) sedation adequacy; (4) procedure duration; (5) vital sign stability; (6) adverse events; and (7) parental satisfaction. Data were collected at baseline and during the procedure.

**Results:**

The mean Comfort-B score was 12.15 ± 2.08 in the study group versus 15.42 ± 2.67 in the control group, with a mean difference of −3.27 points (95% CI –4.02 to −2.52) and a large effect size (Cohen’s d = 1.37, 95% CI 1.03–1.71; *p* < 0.001). The study group also demonstrated significantly better operational compliance (92.68% vs. 79.01%, *p* = 0.012; OR 3.37, 95% CI 1.26–8.98) and overall compliance (93.90% vs. 80.25%, *p* = 0.009; OR 3.79, 95% CI 1.32–10.90). Sedation adequacy with a higher proportion achieving optimal sedation (90.24% vs. 70.37%, *p* = 0.001; OR 3.89, 95% CI 1.63–9.30). Procedure duration was significantly shorter in the study group (12.41 ± 2.26 vs. 15.67 ± 3.12 min, *p* < 0.001; Cohen’s d = 1.20, 95% CI 0.87–1.53). Vital sign stability with significantly smaller fluctuations in heart rate, respiratory rate, and oxygen saturation (all *p* < 0.001; Cohen’s d 1.19–1.87). Parental satisfaction was significantly higher in the study group (93.90% vs. 81.48%, *p* = 0.008; OR 3.79, 95% CI 1.32–10.90).

**Conclusion:**

The multicomponent goal-directed sedation nursing intervention bundle significantly improves comfort, compliance, and sedation adequacy in children undergoing BAL under topical anesthesia with procedural sedation, reduces procedure duration, maintains vital sign stability, and enhances parental satisfaction. Although adverse events were numerically fewer, this difference did not reach statistical significance. This nursing approach provides a safe, effective framework for managing pediatric patients during BAL procedures.

## Introduction

Bronchoalveolar lavage (BAL) is a critical diagnostic and therapeutic procedure for pediatric respiratory diseases, including refractory pneumonia, interstitial lung disease, and airway foreign body aspiration (1). The procedure involves instilling and aspirating sterile saline through a bronchoscope into the alveolar space, allowing for the collection of bronchoalveolar fluid for microbiological, cytological, and immunological analysis (2). While BAL is generally considered safe, it is an invasive procedure that can cause significant discomfort, anxiety, and distress in pediatric patients, potentially compromising procedural success and patient safety (3).

In clinical practice, most BAL procedures in children are performed under topical anesthesia with procedural sedation, as general anesthesia carries additional risks and requires more complex perioperative management (4). However, under local anesthesia alone, children often experience fear, pain, and involuntary movements during the procedure, which can lead to procedural interruptions, increased procedure duration, incomplete sampling, and even procedural failure (5). Furthermore, the distress experienced by children during the procedure can negatively impact their psychological well-being and future willingness to undergo necessary medical procedures (6).

Sedation management during pediatric BAL is therefore of paramount importance. Effective sedation should achieve an optimal balance: sufficient to ensure patient comfort and cooperation, but not so deep as to compromise respiratory function or prolong recovery (7). Traditional sedation approaches often lack individualization, using fixed protocols that may not account for variations in children’s age, disease severity, and anxiety levels. This “one-size-fits-all” approach can result in inadequate sedation (leading to distress and poor cooperation) or excessive sedation (increasing the risk of respiratory depression and other complications) (8).

Goal-directed sedation, originally developed in intensive care settings, represents a paradigm shift from fixed-protocol sedation to individualized, target-driven management (9). In this approach, sedation is titrated to achieve predefined clinical targets (e.g., a specific sedation score, comfort level, or physiological parameter), with ongoing assessment and adjustment (10). This strategy has been shown to reduce the duration of mechanical ventilation, shorten intensive care unit stays, along with improve patient outcomes in critically ill adults (11). However, the application of goal-directed sedation principles in pediatric procedural sedation, particularly for BAL under topical anesthesia with procedural sedation, has received limited attention.

Despite the recognized importance of sedation and comfort management in pediatric BAL, there is a lack of standardized, evidence-based nursing protocols specifically designed for this context. Most existing studies have focused on pharmacological approaches to sedation, with limited attention to the nursing interventions that complement and enhance these strategies (12). Furthermore, the integration of goal-directed principles into nursing care for pediatric procedural sedation remains underexplored.

This study is designed to address this gap by evaluating a goal-directed sedation nursing intervention for children undergoing BAL under topical anesthesia with procedural sedation. We hypothesize that this goal-directed approach will significantly improve children’s comfort and compliance, achieve optimal sedation levels, reduce procedure duration, maintain vital sign stability, decrease adverse events, and enhance parental satisfaction.

## Methods

### Trial registration and protocol

This randomized controlled trial was prospectively registered with the Chinese Clinical Trial Registry (registration number: ChiCTR2400094114) prior to the enrollment of the first participant.

### Study design and participants

This randomized controlled trial was conducted at a tertiary children’s hospital from November 2024 to October 2025. All participants were inpatients admitted to the respiratory department and referred for elective BAL under topical anesthesia with procedural sedation. No ambulatory or outpatient procedures were included. A total of 168 children undergoing BAL under topical anesthesia with procedural sedation were recruited. Inclusion criteria were: (1) aged 3–12 years; (2) scheduled for BAL under local anesthesia for diagnostic or therapeutic purposes; (3) American Society of Anesthesiologists (ASA) physical status I or II; (4) no known allergy to sedative medications. Exclusion criteria were: (1) emergency procedures; (2) previous adverse reaction to sedation; (3) severe cardiopulmonary disease (ASA ≥ III); (4) neurological disorders affecting cooperation; (5) history of psychological trauma or phobia related to medical procedures; and (6) refusal to participate. The study protocol was approved by the Institutional Review Board of Tianjin Children’s Hospital. All procedures were conducted in accordance with the Declaration of Helsinki. Written informed consent was obtained from a parent or legal guardian of each participant before enrollment. In addition, verbal assent was obtained from children aged 6 years and above, in accordance with local ethical requirements.

### Randomization, allocation concealment, and blinding

Participants were randomly assigned to the study group (goal-directed sedation nursing intervention, *n* = 84) or the control group (conventional nursing care, *n* = 84) utilizing a computer-generated random number sequence. An independent biostatistician who was not involved in participant care generated the random allocation sequence using a computer random number generator with a 1:1 allocation ratio and random block sizes of 4 and 6. The sequence was transferred to a research coordinator not otherwise engaged in enrollment or clinical activities. This coordinator prepared sequentially numbered, opaque, sealed envelopes, each containing a card indicating the assigned group. The envelopes were stored in a locked drawer in a secured research office, accessible only to the coordinator.

After a potentially eligible participant was identified and written informed consent obtained by a designated research nurse, the nurse telephoned the coordinator. The coordinator then opened the next envelope in the sequence and communicated the group assignment to the nurse. The enrolling nurse subsequently arranged for the allocated intervention. Owing to the nature of the nursing intervention, blinding of participants, parents, and the nursing staff who delivered the intervention was not feasible. However, the bronchoscopist who rated compliance, the research assistants who collected outcome data, and the statistician who performed the analyses were all blinded to group assignment.

### Sample size calculation

Sample size was calculated based on the primary outcome (mean during-procedure comfort score) in a pilot study (*n* = 20 per group), which showed a mean difference of 2.1 points (standard deviation 1.8) between the two groups. With a two-sided α of 0.05 and power of 0.85, the required sample size was 76 per group. Considering a 10% dropout rate, 168 participants (84 per group) were enrolled.

### Anesthesia and sedation protocol

All procedures were implemented under topical anesthesia with procedural sedation, with the airway anesthetized using topical lidocaine. No premedication or sedative agents (oral, intranasal, or otherwise) were administered prior to intravenous line placement in either group. To minimize pain during venipuncture, topical anesthetic cream was applied to the puncture site; this local measure does not affect the level of sedation. For both groups, the sedation protocol was standardized: midazolam 0.05–0.1 mg/kg was administered intravenously according to body weight before the procedure, with additional doses as needed based on the child’s response. In the study group, sedation was titrated according to goal-directed nursing assessment, while in the control group, sedation was administered according to a fixed protocol without titration based on real-time nursing assessment.

### Intervention

Children in the control group received conventional nursing care, which consisted of: (1) routine pre-procedural education for parents; (2) standard positioning for BAL; (3) basic monitoring of vital signs; and (4) post-procedural observation. In accordance with unit policy, one parent was permitted to remain at the child’s bedside throughout the procedure; however, parents received no coaching or structured guidance on providing comfort or reassurance. No structured comfort assessment or goal-directed sedation titration was performed by nursing staff. Sedation administration was managed by the procedural team based on fixed criteria.

Children in the study group received goal-directed sedation nursing intervention bundle, a structured, multicomponent nursing protocol designed based on goal-directed sedation principles and adapted for pediatric BAL under local anesthesia. The bundle integrated sedation management with comfort-oriented nursing components—including comfort assessment, psychological preparation, distraction, environmental modification, and parental involvement—delivered as a cohesive package by trained nurses. The intervention consisted of four integrated components:

1. Pre-procedural preparation (30–45 min before procedure)

**Comfort assessment**: Baseline comfort level was assessed using the Comfort Behavior Scale (Comfort-B). Individual comfort goals were established in collaboration with the child and parents.

**Psychological preparation**: Age-appropriate explanations of the procedure were provided using visual aids, dolls, or videos. Distraction techniques (music, storytelling, interactive games) were introduced.

**Parental involvement**: Parents were encouraged to stay with the child and were coached on how to provide comfort and reassurance during the procedure.

**Sedation goal setting**: Target sedation level (Ramsay Sedation Scale score 3–4, indicating cooperative and calm with mild sedation) was established for each child based on age, anxiety level, and procedural requirements.

1. During-procedure goal-directed sedation and comfort management

**Continuous comfort monitoring**: Comfort level was assessed every 5 min using the Comfort Behavior Scale (Comfort-B). Sedation was titrated to achieve and maintain the target comfort and sedation goals.

**Goal-directed sedation titration**: Sedation administration was guided by ongoing nursing assessment:

Ramsay score 1–2 (inadequate): Additional sedation considered.

Ramsay score 3–4 (optimal): Maintain current sedation.

Ramsay score 5–6 (excessive): Hold sedation, monitor respiratory status.

**Active comfort interventions**: Based on comfort assessment, nursing interventions were implemented:

Physical comfort: Positioning support, gentle touch, warmth maintenance.

Psychological comfort: Continuous verbal reassurance, distraction (music, storytelling, guided imagery).

Environmental comfort: Dimmed lighting, reduced noise, calm atmosphere.

**Vital sign monitoring**: Heart rate, respiratory rate, blood pressure, and oxygen saturation were continuously monitored and recorded every 5 min. Changes were immediately communicated to the procedural team.

**Parental participation**: Parents were supported to provide comfort and reassurance as appropriate, with guidance from the nursing staff.

1. Post-procedural care

**Immediate comfort assessment**: Comfort level was reassessed immediately after the procedure. Additional comfort interventions were provided as needed.

**Sedation recovery monitoring**: Level of sedation was monitored until return to baseline. Recovery time was recorded.

**Parental feedback**: Parents were given information about post-procedural care and encouraged to report any concerns.

1. Quality assurance

**Nurse training**: All nurses in the study group received 8 h of training on goal-directed sedation principles, comfort assessment, and intervention techniques.

**Competency validation**: Nurses were required to demonstrate competency in comfort assessment and sedation goal setting before participating in the study.

**Intervention fidelity**: A checklist was used to ensure all components of the intervention were implemented as planned.

### Outcome measures

Comfort level was designated as the primary outcome; procedure compliance, sedation adequacy, procedure duration, vital sign stability, adverse events, and parental satisfaction were secondary outcomes.

Comfort level (primary outcome) was assessed using the Comfort Behavior Scale (Comfort-B), a validated observational tool originally developed to assess distress and comfort in pediatric intensive care unit patients under mechanical ventilation and sedation. The scale comprises 6 behavioral items—alertness, calmness/agitation, respiratory response (in ventilated patients) or crying (in spontaneously breathing patients), physical movement, facial tension, and muscle tone—each scored on a 5-point scale (1–5), yielding a total score ranging from 6 to 30. Lower scores indicate greater comfort (optimal sedation typically corresponds to scores of 11–17). The Comfort-B has established inter-rater reliability (intraclass correlation coefficient 0.96–0.98) and internal consistency (Cronbach’s α 0.84–0.90) in the PICU population and has been validated against the Numeric Rating Scale for pain and the Sedation–Agitation Scale in mechanically ventilated and non-ventilated children (13). Although the Comfort-B scale has well-established reliability and validity in the PICU setting, it has not been formally validated for assessing comfort during pediatric procedural sedation. In the spontaneously breathing children undergoing BAL, the item “respiratory response” was replaced by “crying” according to the scale manual. Assessments were performed at baseline, during the procedure (every 5 min), and immediately post-procedure. The mean during-procedure score was used for analysis.

Compliance was assessed by the bronchoscopist using a structured, locally developed observational rating scale consisting of two components: Operational compliance: Ability to remain still during critical procedural steps (scoring: 0 = poor, frequent movement; 1 = fair, occasional movement; 2 = good, minimal movement; 3 = excellent, no movement). Scores ≥2 were classified as compliant. Overall compliance: Global assessment of cooperation (scoring: 0 = refused/uncooperative; 1 = cooperative with difficulty; 2 = cooperative with minor difficulty; 3 = fully cooperative). Scores ≥2 were classified as compliant. This scale was designed for the present study based on clinical experience and descriptions of cooperative behavior in previous pediatric procedural studies. Clear behavioral anchors for each level were described in a written scoring guide. The bronchoscopist who rated compliance was blinded to group allocation and received specific instruction on the scoring definitions before the study. To reduce inter-rater variability, all assessments were performed by a single experienced bronchoscopist who had >10 years of experience in pediatric bronchoscopy. However, the scale has not undergone formal psychometric validation, and reliability metrics (e.g., inter-rater or test–retest reliability) are not available.

Sedation level was assessed using the Ramsay Sedation Scale: 1 = anxious/agitated; 2 = cooperative/oriented/tranquil; 3 = responsive to commands only; 4 = brisk response to light glabellar tap; 5 = sluggish response; 6 = no response. Optimal sedation was defined as Ramsay score 3–4 (cooperative, calm, responsive to commands). The Ramsay scale is the most widely used sedation scale for invasive procedures in pediatrics; a recent prospective validation study demonstrated its validity, reliability (intra-observer agreement *ρ* = 0.884; interobserver intraclass correlation coefficient = 0.94), and internal consistency (α = 0.91) for monitoring sedation in pediatric patients undergoing invasive procedures (14). Assessments were performed before sedation, every 5 min during the procedure, and post-procedure until recovery. For the participant-level analysis of sedation adequacy, each child was assigned to a single Ramsay category (1–2, 3–4, or 5–6) based on the most frequent score recorded during the bronchoscopy procedure, yielding mutually exclusive groups whose totals equal the number of participants analyzed.

Procedure duration was defined as the time from bronchoscope insertion to removal, measured in minutes.

Vital signs including heart rate (HR), respiratory rate (RR), systolic blood pressure (SBP), diastolic blood pressure (DBP), and oxygen saturation (SpO₂) were monitored continuously and recorded every 5 min. Stability was assessed by calculating the maximum change from baseline during the procedure.

Adverse events were defined as any untoward medical occurrence during or immediately after the procedure, including: respiratory depression (SpO₂ < 90%); bradycardia (HR < age-specific lower limit); hypotension (SBP < age-specific lower limit); agitation requiring physical restraint; procedural interruption; and vomiting/aspiration. All adverse events were recorded and graded for severity. Severity was classified as: mild (transient, not requiring any specific intervention beyond observation); moderate (requiring an intervention such as supplemental oxygen, temporary interruption of the procedure, or additional medication); or severe (requiring immediate termination of the procedure, escalation of care, or resulting in permanent sequelae). The type and duration of any intervention administered in response to an adverse event were documented.

Parental satisfaction was assessed using a 10-item questionnaire covering: (1) overall satisfaction with care; (2) perceived child comfort; (3) perceived child cooperation; (4) communication from nursing staff; (5) support provided to parents; (6) quality of sedation management; (7) procedure experience; (8) recovery experience; (9) willingness to recommend; and (10) overall impression. Each item was scored on a 5-point Likert scale (1 = very dissatisfied, 5 = very satisfied). Scores ≥4 were classified as satisfied. The questionnaire was adapted from a previously published instrument used in pediatric procedural sedation studies (15). The adapted version was reviewed for face validity by a panel of three pediatric nursing experts and pilot-tested with 10 parents of children who had previously undergone BAL to confirm clarity and relevance. Internal consistency in the present sample was acceptable (Cronbach’s α = 0.87). However, the questionnaire has not undergone formal construct validation, and its responsiveness to changes in the quality of procedural care has not been established.

### Data collection

Data were obtained by trained research assistants who were blinded to group allocation. Pre-procedural data (baseline comfort, demographics, clinical characteristics) were collected before the intervention. During-procedure data (compliance, comfort scores, sedation scores, vital signs, procedure duration, adverse events) were recorded by the research assistants. Post-procedural data (parental satisfaction) were collected within 24 h after the procedure using self-administered questionnaires. Double data entry was performed to ensure accuracy. In addition, the research assistant documented for each procedure whether a parent was present in the room (yes/no) and, if so, whether the parent actively provided comfort to the child. This information was used to verify comparability between groups.

### Statistical analysis

Statistical analysis was implemented using SPSS version 26.0. Continuous data were expressed as mean ± standard deviation (SD), and categorical data as frequencies and percentages. Normality of continuous data was assessed using the Shapiro–Wilk test. For normally distributed data, independent *t*-tests were used for between-group comparisons. For non-normally distributed data, Mann–Whitney U tests were used. Categorical data were analyzed using the chi-square test or Fisher’s exact test as appropriate. Repeated measures ANOVA was used to compare changes over time between groups. Effect sizes were calculated to quantify the magnitude of differences. For continuous outcomes, Cohen’s d with 95% confidence intervals (CIs) was computed, where d = 0.2, 0.5, and 0.8 correspond to small, medium, and large effects, respectively. For categorical outcomes, odds ratios (OR) with 95% CIs and Cramér’s V were reported. Since a single primary outcome was specified, no adjustment for multiplicity was applied to secondary outcomes; *p* values for secondary endpoints are reported as nominal, and these results should be interpreted as exploratory. A two-sided *p* < 0.05 was considered statistically significant. The primary analysis was based on the per-protocol population, consisting of all randomized participants who completed the assigned procedure with no major protocol deviations and had complete outcome data. Five participants (2 study, 3 control) were excluded due to protocol deviations that resulted in completely missing data; no imputation was performed. Accordingly, all proportions, percentages, and tests of association use denominators of 82 for the study group and 81 for the control group.

## Results

### Participant flow

**Figure 1:**
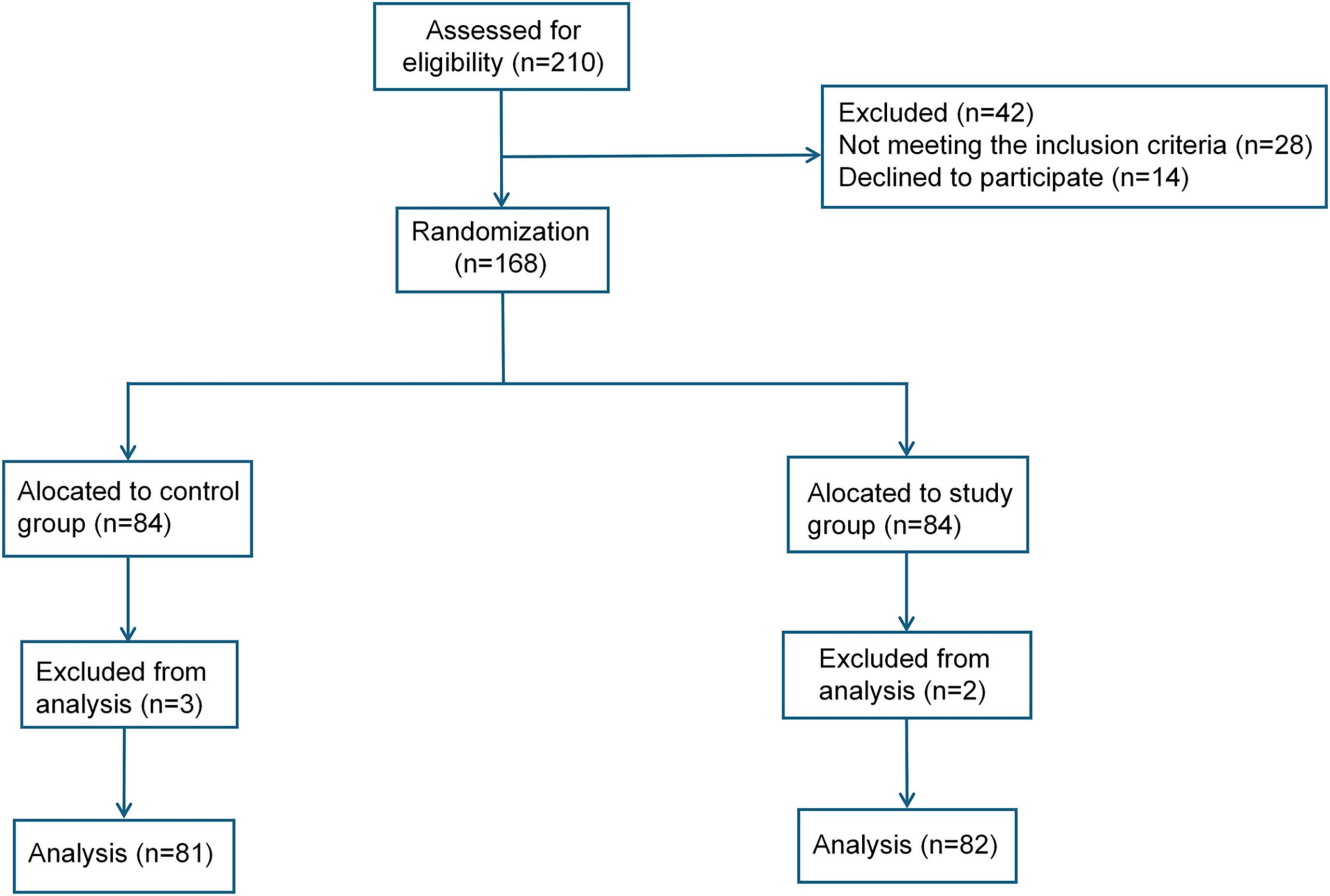
Participant flow diagram.

Between November 2024 and October 2025, a total of 210 children scheduled for elective bronchoscopy were screened for eligibility. Of these, 42 were excluded (28 did not meet all inclusion criteria, 14 declined to participate). The remaining 168 children were randomly assigned to the study group (*n* = 84) and the control group (*n* = 84). During the study period, 2 participants in the study group and 3 in the control group were excluded from analysis due to protocol deviations that resulted in incomplete data, leaving 82 participants in the study group and 81 in the control group for the final analysis. The flow of participants through the trial is depicted in Figure 1. All subsequent analyses were conducted on this complete-case sample.

### Baseline characteristics

Baseline characteristics were comparable between the two groups, with no significant differences in age, sex, weight, ASA status, underlying condition, or baseline comfort scores (all *p* > 0.05), indicating successful randomization (Table 1). Both groups had a 100% rate of parental presence during the procedure, and no child underwent the procedure without a parent at the bedside. Accordingly, parental presence was not a confounding factor in the analysis.

**Table 1:** Baseline characteristics of participants.

Characteristic	Study group (*n* = 82)	Control group (*n* = 81)	*t*/χ^2^	*p*-value
Age (years, mean ± SD)	6.45 ± 2.34	6.67 ± 2.21	−0.623	0.534
Sex, *n* (%)			0.234	0.629
Male	45 (54.88)	42 (51.85)		
Female	37 (45.12)	39 (48.15)		
Weight (kg, mean ± SD)	22.34 ± 5.67	23.01 ± 5.89	−0.745	0.457
ASA status, *n* (%)			0.123	0.726
I	58 (70.73)	56 (69.14)		
II	24 (29.27)	25 (30.86)		
Underlying condition, *n* (%)			0.456	0.928
Refractory pneumonia	51 (62.20)	49 (60.49)		
Interstitial lung disease	18 (21.95)	19 (23.46)		
Foreign body aspiration	8 (9.76)	7 (8.64)		
Other	5 (6.10)	6 (7.41)		

### Primary outcome: comfort level

**Figure 2:**
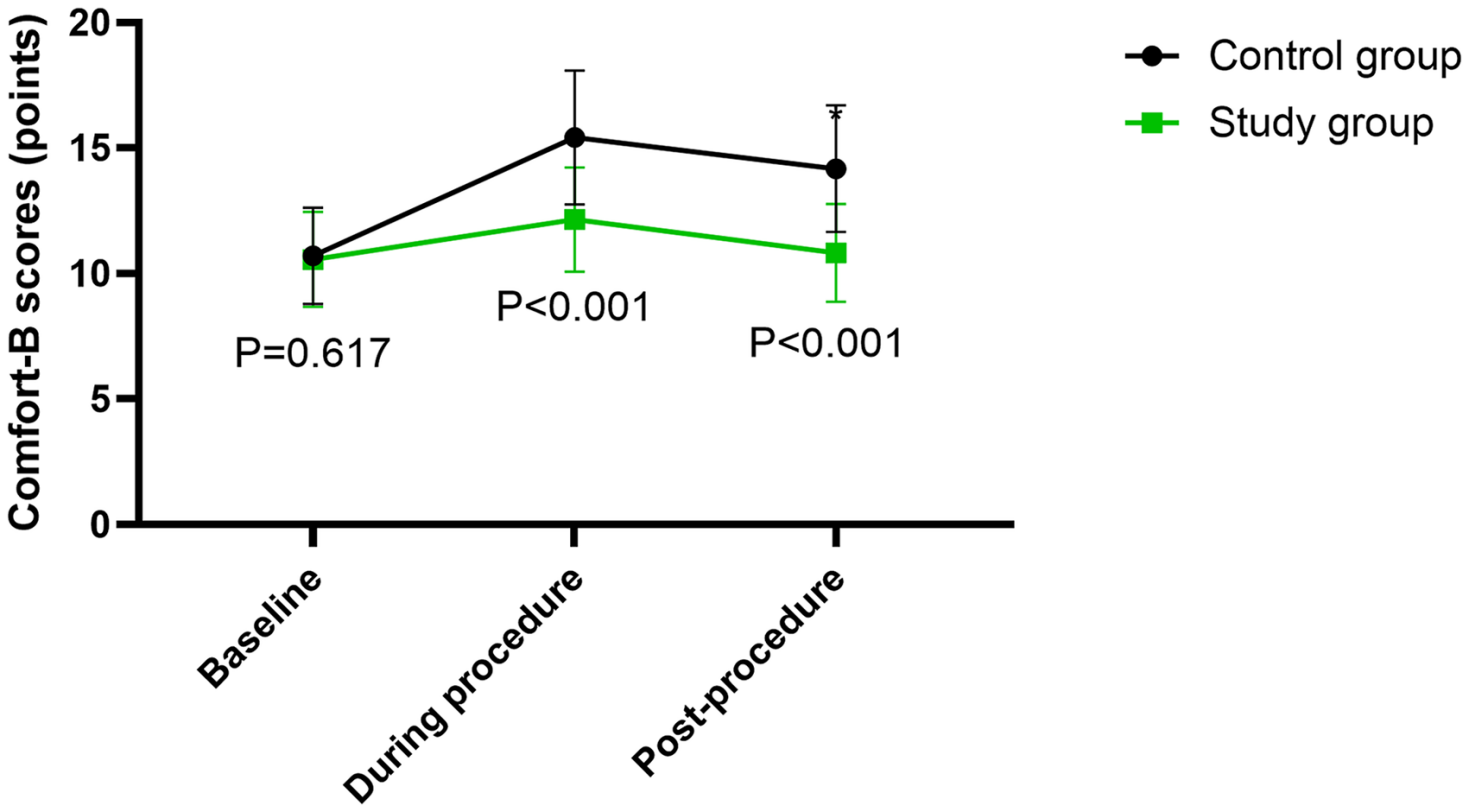
Comparison of comfort level between the two groups. Bars represent mean ± standard deviation. Individual data points are overlaid. *p* values were derived from independent *t*-tests.

The study group achieved significantly lower Comfort-B scores (indicating greater comfort) during the procedure and post-procedure compared to the control group (Figure 2). At baseline, Comfort-B scores were comparable between groups (study: 10.56 ± 1.89; control: 10.71 ± 1.92; mean difference −0.15, 95% CI –0.74 to 0.44; Cohen’s d = 0.08; *p* = 0.617), confirming successful randomization. During the procedure, the mean Comfort-B score was 12.15 ± 2.08 in the study group versus 15.42 ± 2.67 in the control group, with a mean difference of −3.27 points (95% CI –4.02 to −2.52) and a large effect size (Cohen’s d = 1.37, 95% CI 1.03–1.71; *p* < 0.001). This significant difference persisted post-procedure (study: 10.83 ± 1.95; control: 14.18 ± 2.53; mean difference −3.35, 95% CI –4.07 to −2.63; Cohen’s d = 1.49, 95% CI 1.14–1.84; *p* < 0.001).

### Secondary outcomes

#### Procedure compliance

The study group demonstrated significantly better compliance compared to the control group. Operational compliance (scores ≥2) was achieved in 92.68% (76/82) of the study group vs. 79.01% (64/81) of the control group (χ^2^ = 6.28, *p* = 0.012), with an OR of 3.37 (95% CI 1.26–8.98) and a Cramér’s V of 0.20. Overall compliance was achieved in 93.90% (77/82) of the study group vs. 80.25% (65/81) of the control group (χ^2^ = 6.77, *p* = 0.009), with an OR of 3.79 (95% CI 1.32–10.90) and a Cramér’s V of 0.20 (Table 2).

**Table 2:** Comparison of procedure compliance.

Outcome	Study group (*n* = 82)	Control group (*n* = 81)	χ^2^	*p*-value
Operational compliance (scores ≥2)	76 (92.68%)	64 (79.10%)	6.28	0.012
Overall compliance (scores ≥2)	77 (93.90%)	65 (80.25%)	6.77	0.009

#### Sedation adequacy

Sedation adequacy was significantly better in the study group. The proportion of children achieving optimal sedation (Ramsay score 3–4) was 90.24% (74/82) in the study group vs. 70.37% (57/81) in the control group (χ^2^ = 9.97, *p* = 0.002), with an OR of 3.89 (95% CI 1.63–9.30) and a Cramér’s V of 0.25. Inadequate sedation (Ramsay 1–2) was observed in 7.32% (6/82) of the study group versus 25.93% (21/81) of the control group, while excessive sedation (Ramsay 5–6) was observed in 2.44% (2/82) versus 3.70% (3/81), respectively (Table 3).

**Table 3:** Comparison of sedation adequacy.

Ramsay score	Study group (*n* = 82)	Control group (*n* = 81)	χ^2^/Fisher’s exact	*p*-value
1–2 (inadequate)	6 (7.32%)	21 (25.93%)	10.16	0.001
3–4 (optimal)	74 (90.24%)	57 (70.37%)	9.97	0.002
5–6 (excessive)	2 (2.44%)	3 (3.70%)		0.676

#### Procedure duration

**Figure 3:**
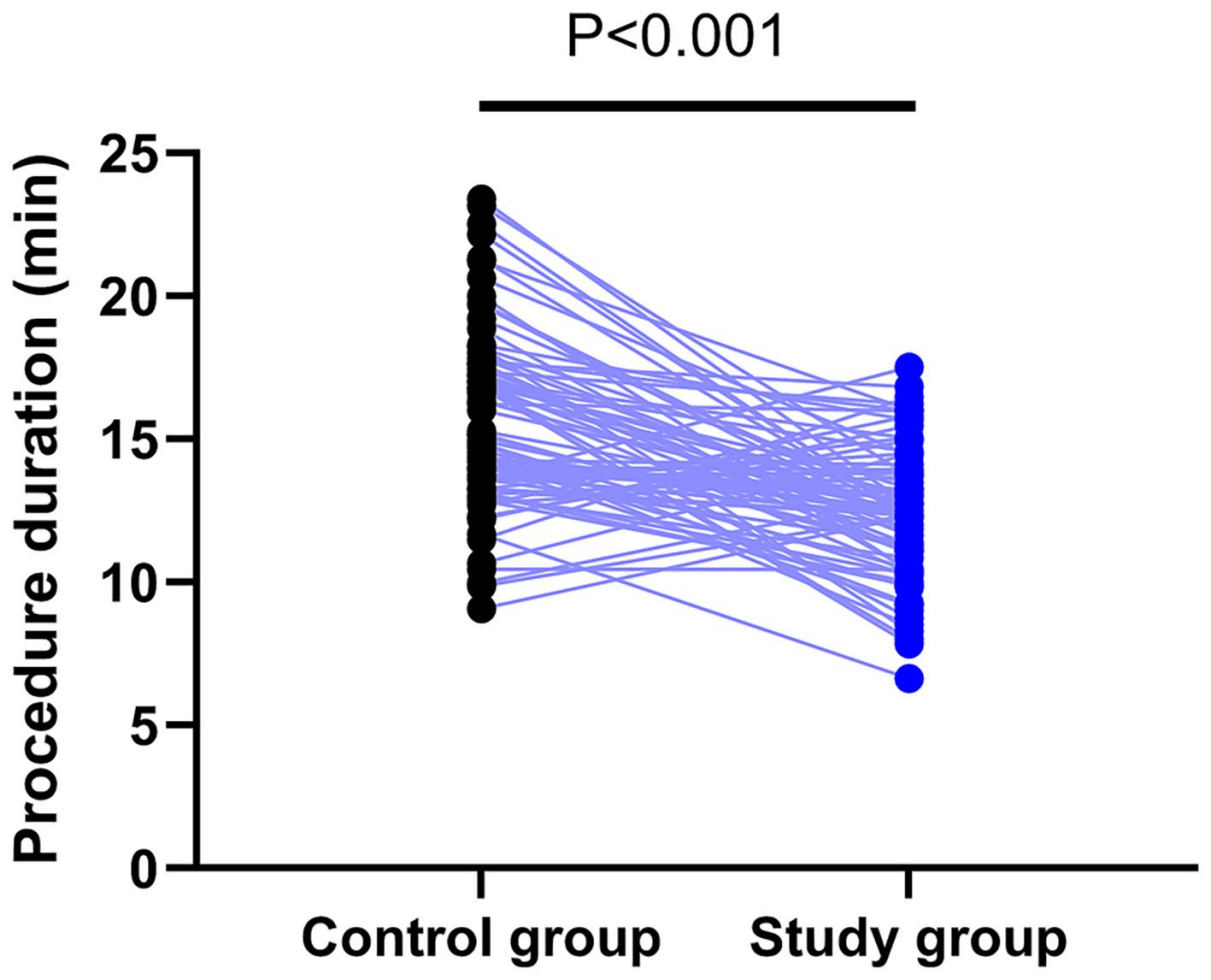
Comparison of procedure duration between the two groups. Bars represent mean ± standard deviation. Individual data points are overlaid. *p* values were derived from independent *t*-tests.

Procedure duration was significantly shorter in the study group (12.41 ± 2.26 min) compared to the control group (15.67 ± 3.12 min), with a mean difference of 3.33 min (*p* < 0.001, Cohen’s d = 1.20, 95% CI 0.87–1.53, Figure 3).

#### Vital sign stability

**Figure 4:**
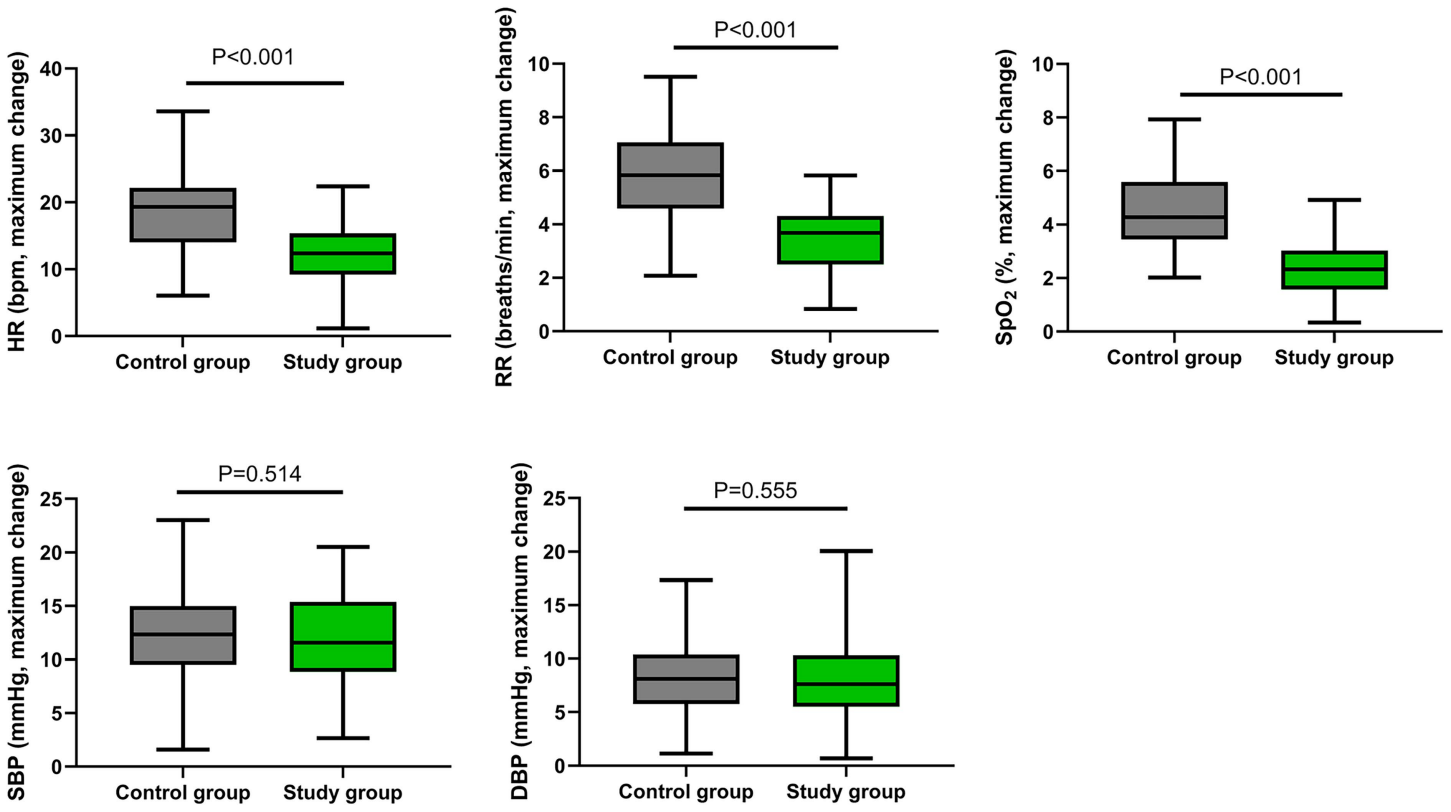
Comparison of vital sign stability between the two groups. Bars represent mean ± standard deviation. Individual data points are overlaid. *p* values were derived from independent *t*-tests.

Vital sign stability was better maintained in the study group. The maximum changes from baseline during the procedure were significantly smaller in the study group for HR (12.34 ± 4.56 vs. 18.45 ± 5.67 bpm, *p* < 0.001, Cohen’s d = 1.19, 95% CI 0.86–1.52), RR (3.45 ± 1.23 vs. 5.67 ± 1.89 breaths/min, *p* < 0.001, Cohen’s d = 1.39, 95% CI 1.06–1.73), and SpO₂ (2.34 ± 1.02 vs. 4.56 ± 1.34%, *p* < 0.001, Cohen’s d = 1.87, 95% CI 1.50–2.23). Changes in blood pressure were not significantly different between groups (*p* = 0.514 and *p* = 0.555) (Figure 4).

#### Adverse events

The number of participants experiencing at least one adverse event was 5 (6.10%) in the study group and 12 (14.81%) in the control group. No participant experienced more than one type of adverse event. The between-group difference was not statistically significant (Fisher’s exact test, *p* = 0.072). The odds of experiencing an adverse event were numerically reduced (OR 0.37, 95% CI 0.12–1.11; Cramér’s V = 0.14). Specific adverse events are detailed in Table 4.

**Table 4:** Comparison of adverse events.

Adverse event	Study group (*n* = 82)	Control group (*n* = 81)	*p*-value
Respiratory depression (SpO₂ < 90%)	1 (1.22%)	3 (3.70%)	
Bradycardia	0 (0%)	2 (2.47%)	
Agitation requiring restraint	2 (2.44%)	5 (6.17%)	
Procedural interruption	2 (2.44%)	6 (7.41%)	
Vomiting/aspiration	1 (1.22%)	2 (2.47%)	
Total adverse events	5 (6.10%)	12 (14.81%)	0.072

Data are number of participants who experienced at least one event in each listed category. No participant experienced more than one type of adverse event. Fisher’s exact test for “any adverse event”: *p* = 0.072 (two-sided).

All adverse events were graded as mild or moderate; no severe adverse events occurred. Respiratory depression (SpO₂ < 90%) was managed with oxygen supplementation via nasal cannula and brief interruption of the procedure, with all cases recovering within 2 min. The two cases of bradycardia in the control group resolved spontaneously after temporary interruption of the procedure; no atropine or other pharmacological intervention was required. Agitation requiring physical restraint was managed with additional sedation (midazolam 0.05 mg/kg IV) and parental reassurance. Procedural interruptions were transient (all < 3 min), and the BAL procedure was completed in all cases. The episode of vomiting occurred after the procedure and was managed with suctioning and lateral positioning; no aspiration was observed. No participant required escalation of care, unplanned admission, or prolonged hospitalization due to adverse events.

#### Parental satisfaction

Parental satisfaction was significantly higher in the study group. The satisfaction rate was 93.90% (77/82) in the study group vs. 81.48% (66/81) in the control group (χ^2^ = 6.96, *p* = 0.008), with an OR of 3.79 (95% CI 1.32–10.90) and a Cramér’s V of 0.21 (Table 5).

**Table 5:** Comparison of parental satisfaction.

Group	*n*	Satisfied	Not satisfied	Satisfaction rate (%)
Study group	82	77	5	93.90
Control group	81	66	15	81.48
χ^2^				6.96
*p*-value				0.008

## Discussion

This randomized controlled trial provides strong evidence that a structured, multicomponent goal-directed sedation nursing intervention bundle is associated with significantly improved comfort, compliance, and sedation adequacy in children undergoing bronchoalveolar lavage under topical anesthesia with procedural sedation. These findings, while requiring confirmation in larger multicenter studies, suggest a bundled, goal-driven approach to sedation and comfort management yields superior outcomes compared to conventional nursing care.

The study group achieved significantly lower Comfort-B scores throughout the procedure, indicating greater comfort and supporting the hypothesis that the intervention improves procedural comfort. The goal-directed approach ensured that comfort was continuously assessed and actively managed, rather than simply monitored. By setting individual comfort goals and titrating interventions accordingly, nurses were able to proactively address emerging distress before it escalated. This finding is clinically significant, as even modest improvements in comfort can translate into meaningful reductions in distress and anxiety (16).

The superior compliance observed in the study group (93.90% vs. 80.25%) is likely a direct consequence of improved comfort and sedation adequacy. Children who are comfortable, calm, and appropriately sedated are better able to follow instructions and remain still during critical procedural steps. This finding is in accordance with previous reports demonstrating that effective sedation and comfort management improve procedural success rates in pediatric populations (17).

The goal-directed sedation approach resulted in a significantly higher proportion of children achieving optimal sedation (Ramsay score 3–4) compared to conventional care (90.24% vs. 70.37%). This finding highlights the limitations of fixed-protocol sedation, which often fails to account for individual variation in drug response and procedural requirements. In contrast, goal-directed sedation, guided by ongoing nursing assessment, allows for real-time titration to achieve and maintain the desired sedation level. Importantly, the goal-directed approach also reduced the incidence of inadequate sedation (Ramsay 1–2) from 25.93% in the control group to 7.32% in the study group. Inadequate sedation is particularly problematic during pediatric BAL, as uncooperative children may require physical restraint, increasing procedural risk and psychological distress (18). Similarly, excessive sedation (Ramsay 5–6) was less common in the study group, although the difference had no statistical significance.

Procedure duration was significantly shorter in the study group, representing a 21% reduction in procedural time. This finding has important clinical implications, as shorter procedures reduce the duration of airway manipulation, minimize the risk of complications, and improve operating room efficiency. The shorter procedure duration likely reflects the combined effects of better compliance (reducing the need for repeated attempts) and more stable vital signs (reducing the need for pauses to manage adverse events).

Better maintenance of vital sign stability in the study group, particularly for HR, RR, and SpO₂, indicates that goal-directed sedation nursing intervention contributes to physiological stability during the procedure. Excessive sympathetic activation associated with pain and distress can cause tachycardia, tachypnea, and increased oxygen consumption, potentially compromising oxygen delivery to vital organs (19). By maintaining comfort and optimal sedation, the goal-directed approach helped mitigate these physiological stress responses. All vital sign differences exhibited large effect sizes (Cohen’s d 1.19–1.87), underscoring the clinical relevance of the intervention for maintaining physiological stability. The absence of significant differences in blood pressure changes between groups suggests that the sedation levels achieved in both groups were generally safe from a hemodynamic perspective. However, the greater stability in oxygenation (SpO₂) in the study group is clinically important, as hypoxemia is a common concern during BAL in children.

Although the incidence of total adverse events did not differ significantly between groups (*p* = 0.072), the numerically lower rate in the study group (6.10% vs. 14.81%) suggests a favorable safety profile. Events such as procedural interruptions and agitation requiring restraint were observed less frequently with goal-directed sedation, which likely reduces distress- and movement-related procedural risks. The lack of statistical significance may reflect insufficient power for rare events; a larger, multicenter sample would be needed to confirm whether the intervention truly reduces adverse event rates.

The high level of parental satisfaction in the study group (93.90% vs. 81.48%) reflects the holistic benefits of goal-directed sedation nursing intervention. Parents observed their children being more comfortable and cooperative, experienced better communication and support from nursing staff, and felt more involved in their child’s care. Parental satisfaction is increasingly recognized as an important quality indicator in pediatric procedural care, as satisfied parents are more likely to adhere to follow-up recommendations and report positive experiences to others.

The effectiveness of goal-directed sedation nursing intervention bundle can be attributed to several mechanisms: First, individualized goal setting ensures that sedation and comfort management are tailored to each child’s unique needs, rather than applying a one-size-fits-all protocol. This accounts for variations in age, developmental stage, anxiety level, and procedural requirements. Second, continuous assessment and titration allow for real-time adjustment of interventions based on the child’s response, preventing both under- and over-sedation. The use of validated assessment tools (Comfort-B, Ramsay Scale) provides objective, reproducible measures to guide clinical decisions. Third, integrated comfort interventions address multiple dimensions of comfort—physical, psychological, and environmental—creating a holistic approach that complements pharmacological sedation. The involvement of parents as active participants in comfort provision leverages the child’s existing support system. Fourth, proactive rather than reactive management anticipates distress before it escalates, reducing the need for more intensive interventions. Regular assessment every 5 min allows early detection of emerging distress and timely intervention. Fifth, the intervention, by equipping nurses with specialized training and structured assessment tools, may facilitate a more active nursing role in sedation and comfort management within their existing scope of practice. It is important to note that the observed benefits likely reflect the synergistic effects of the full bundle—combining structured sedation titration with active comfort interventions and parental involvement—rather than any single component in isolation. The bundled design is consistent with contemporary approaches to complex nursing interventions, where multiple interacting components are delivered together to achieve a clinical goal.

## Clinical implications

The findings of this study have several important clinical implications: First, the goal-directed sedation nursing intervention bundle is a promising approach that, if its effects are confirmed in future studies, may provide a structured framework for managing pediatric patients undergoing BAL under topical anesthesia with procedural sedation. The approach can be readily implemented in clinical practice with appropriate nursing training. Second, the intervention has the potential to empower registered nurses to take an active role in sedation and comfort management through systematic assessment, goal-directed monitoring, and proactive comfort interventions, enhancing their contribution to procedural success and patient safety. This represents an expansion of the registered nurse’s clinical decision-making role in procedural sedation care within their existing scope of practice, moving beyond passive implementation of fixed protocols to an active, assessment-driven model of nursing care. Third, the shorter procedure duration and numerically fewer adverse events suggest that the intervention may contribute to resource savings; however, formal cost-effectiveness analysis was not conducted and would be needed to confirm this. Fourth, the high parental satisfaction associated with the intervention may improve the overall patient experience and increase parental trust in healthcare providers.

## Limitations and future research

Several limitations should be acknowledged. First, the study was conducted at a single institution, which may limit generalizability to other settings with different patient populations and practice patterns. Second, blinding of participants and nursing staff to group allocation was not feasible given the nature of the intervention, although outcome assessors were blinded. Third, the relatively short follow-up period did not allow assessment of long-term psychological outcomes or recurrence of fear of medical procedures. Fourth, the study did not evaluate cost-effectiveness, which would be important for resource allocation decisions. Fifth, the sample size, while adequate for the primary outcome, may not have been sufficient to detect differences in rare adverse events. Sixth, the Ramsay Sedation Scale, although recently validated for monitoring sedation during invasive procedures under deep sedation in children, was developed originally for adult intensive care patients; it uses subjective clinical assessment, which may introduce observer variability despite standardized training, and has limited formal validation specifically in the pediatric moderate procedural sedation context. Seventh, the compliance assessment tool used in this study was developed locally and has not undergone formal psychometric validation, which may introduce observer bias and limit the generalizability of these findings. Eighth, the Comfort Behavior Scale (Comfort-B), although extensively validated for monitoring distress in the PICU setting, has not been formally validated for assessing comfort during pediatric procedural sedation in spontaneously breathing children, potentially affecting the accuracy of the comfort assessment. Ninth, the compliance assessment tool was developed locally and lacks formal psychometric validation, and the parental satisfaction questionnaire, while showing acceptable internal consistency, has not been subjected to construct validation; both may limit the reliability of these measurements. Tenth, while a single primary outcome was prespecified, multiple secondary outcomes were tested without adjustment for multiplicity; hence, significant results for secondary endpoints may be inflated, and these findings should be considered exploratory.

Future research should focus on: (1) multi-center studies to establish external validity; (2) long-term follow-up to assess psychological outcomes and recurrence of fear of medical procedures; (3) cost-effectiveness analysis to inform resource allocation; (4) exploration of the intervention’s applicability to other pediatric procedures; (5) investigation of optimal nurse training models for implementing goal-directed sedation approaches; (6) development and validation of pediatric-specific sedation scales suitable for moderate procedural sedation in children, to address the limitations of scales originally designed for adult intensive care; (7) development and psychometric testing of structured, validated instruments for assessing procedural compliance in children undergoing invasive procedures; and (8) validation of comfort assessment tools, such as the Comfort-B, specifically for procedural sedation contexts in pediatrics, to ensure accurate measurement of comfort in non-intubated children undergoing brief invasive procedures.

## Conclusion

In this randomized controlled trial, a multicomponent goal-directed sedation nursing intervention bundle was associated with significantly better comfort, compliance, and sedation adequacy in children undergoing bronchoalveolar lavage under topical anesthesia with procedural sedation. The intervention was also associated with shorter procedure duration, greater vital sign stability, and higher parental satisfaction. The safety profile was comparable between groups. These findings suggest that the bundled intervention is a promising approach for managing pediatric patients during BAL procedures. Further multicenter studies with longer follow-up and validated outcome measures are warranted to confirm these results before the intervention can be recommended for routine clinical practice.

## Data Availability

The datasets presented in this study can be found in online repositories. The names of the repository/repositories and accession number(s) can be found in the article/supplementary material.

## References

[1] Zhang H Deng D Li S Ren J Huang W Liu D . Bronchoalveolar lavage fluid assessment facilitates precision medicine for lung cancer. Cancer Biol Med. (2023) 21:230–51. doi: 10.20892/j.issn.2095-3941.2023.0381, 38164737 PMC10976328

[2] Pogoriler J. Bronchoalveolar lavage: cytology. In: Goldfarb S, Piccione J, editors. Diagnostic and Interventional Bronchoscopy in Children. Cham: Springer International Publishing (2021) p. 69–79. doi: 10.1007/978-3-030-54924-4_8

[3] Pavi I Radi M Golub RA Karai DE Hojsak I. Bronchoalveolar lavage findings in children with chronic unexplained cough. Eur Respir J. (2023) 62:PA1640. doi: 10.1183/13993003.congress-2023.PA1640

[4] Branch of Pediatric Critical Care Physicians, Chinese Medical Association; Neonatologists Branch of Chinese Medical Association; Gansu Provincial Maternal and Child Health Hospital/Gansu Provincial Central Hospital/Gansu Pediatric Clinical Medical Research Center; Center for Evidence-Based Medicine, School of Basic Medicine, Lanzhou University/WHO Guidelines for Practice and Knowledge Transformation Cooperation Center/Gansu Province Medical Guideline Technology Center. Clinical practice guidelines for bronchoalveolar lavage in Chinese children (2024). Zhongguo Dang Dai Er Ke Za Zhi. (2024) 26:1–13. doi: 10.7499/j.issn.1008-8830.2308072 38269452 PMC10817737

[5] Sun R Zhang Y Cheng F Zhao Y. Effect of pulmonary rehabilitation nursing approach with integrated traditional Chinese and western medicine in children with Mycoplasma pneumonia after Bronchoalveolar lavage. Pak J Med Sci. (2025) 41:1105–9. doi: 10.12669/pjms.41.4.9787, 40290258 PMC12022583

[6] Lin WC Qin Y Chun X Huang RL Chen RS Zhang D. Perioperative Care of Children with acute exogenous lipoid pneumonia submitted to BAL/FB. Int J Gen Med. (2021) 14:8383–8. doi: 10.2147/IJGM.S339118, 34819746 PMC8608239

[7] Krylovetskaya MA Gusarova O Savosin RS Valiev TT Kuvshinov YP Grigorievskaya ZV . Bronchoalveolar lavage in pediatric oncohematology. Medical Alphabet. (2022) 31:46–49. doi: 10.33667/2078-5631-2022-31-46-49

[8] Jin QQ Cai WC Zhou YF Zhang YT Chen G Xu MT . Comparison of a ready-to-use intranasal dexmedetomidine spray with traditional intranasal dexmedetomidine drops for sedation in preschool children: a prospective, randomized, controlled study. Front Pharmacol. (2025) 16:1528612. doi: 10.3389/fphar.2025.1528612, 39917619 PMC11799867

[9] Lu S Song H Lin Y Song B Lin S. A randomized controlled trial investigating the impact of early goal-directed sedation dominated by dexmedetomidine on cerebral oxygen metabolism and inflammatory mediators in patients with severe brain injury. Neurol Sci. (2025) 46:1741–50. doi: 10.1007/s10072-024-07916-8, 39673043

[10] Sun X Chen T Wei L Sun L Li X Liang X. Early goal-directed sedation with dexmedetomidine is associated with lower delirium rate in mechanically ventilated patients. BMC Anesthesiol. (2026) 26:130. doi: 10.1186/s12871-026-03651-z, 41593516 PMC12917986

[11] Deng P Hao L Deng Y Yao R Cao Y. Pre-emptive remifentanil alleviates pain associated with tracheal suctioning in patients under mechanical ventilation and goal-directed sedation: a randomized controlled feasibility trial. Int J Nurs Pract. (2022) 28:e12915. doi: 10.1111/ijn.12915, 33403734

[12] Curatola A D'Agostin M Favaretto E Vittori G Vidonis V Strajn T . Nurses' perceptions of the quality of procedural sedation in children comparing different pharmacological regimens. Children Basel. (2022) 9:1068. doi: 10.3390/children9071068, 35884052 PMC9315654

[13] Ista E van Dijk M Tibboel D de Hoog M. Assessment of sedation levels in pediatric intensive care patients can be improved by using the COMFORT "behavior" scale. Pediatr Crit Care Med. (2005) 6:58–63. doi: 10.1097/01.PCC.0000149318.40279.1A, 15636661

[14] Lozano-Díaz D Valdivielso Serna A Garrido Palomo R Arias-Arias Á Tárraga López PJ Martínez GA. Validation of the Ramsay scale for invasive procedures under deep sedation in pediatrics. Paediatr Anaesth. (2021) 31:1097–104. doi: 10.1111/pan.14248, 34173295

[15] Monsereenusorn C Malaithong W Lertvivatpong N Photia A Rujkijyanont P Traivaree C. The efficacy and safety of midazolam with fentanyl versus midazolam with ketamine for bedside invasive procedural sedation in pediatric oncology patients: a randomized, double-blinded, crossover trial. Pediatr Hematol Oncol. (2022) 39:681–96. doi: 10.1080/08880018.2022.2055685, 36239702

[16] Comte A Szymanska M Monnin J Moulin T Nezelof S Magnin E . Neural correlates of distress and comfort in individuals with avoidant, anxious and secure attachment style: an fMRI study. Attach Hum Dev. (2024) 26:1–23. doi: 10.1080/14616734.2024.2384393, 39093338

[17] Jordan KS Steelman SH. Implementing safe and effective pediatric procedural sedation in the emergency department. Adv Emerg Nurs J. (2021) 43:293–302. doi: 10.1097/TME.0000000000000380, 34699418

[18] Edmunds KJ Byczkowski T Frey M Boyd S Caruso M Zhang Y . Risk factors for inadequate sedation after endotracheal intubation in the pediatric emergency department. Am J Emerg Med. (2022) 56:15–20. doi: 10.1016/j.ajem.2022.03.002, 35344822

[19] Klaas KM Fischer PR Segner S Tsai Owens M Fahrenkamp A Geske J . Excessive postural tachycardia and postural orthostatic tachycardia syndrome in youth: associations with distress, impairment, health behaviors, and medication recommendations. J Child Neurol. (2022) 37:599–608. doi: 10.1177/08830738221078410, 35585700 PMC13717731

